# An adult-based insulin resistance genetic risk score associates with insulin resistance, metabolic traits and altered fat distribution in Danish children and adolescents who are overweight or obese

**DOI:** 10.1007/s00125-018-4640-0

**Published:** 2018-05-31

**Authors:** Anne-Sofie Graae, Mette Hollensted, Julie T. Kloppenborg, Yuvaraj Mahendran, Theresia M. Schnurr, Emil Vincent R. Appel, Johanne Rask, Tenna R. H. Nielsen, Mia Ø. Johansen, Allan Linneberg, Marit E. Jørgensen, Niels Grarup, Haja N. Kadarmideen, Birgitte Holst, Oluf Pedersen, Jens-Christian Holm, Torben Hansen

**Affiliations:** 1 Section for Metabolic Receptology, Novo Nordisk Foundation Center for Basic Metabolic Research, Faculty of Health and Medical Sciences, University of Copenhagen, Copenhagen, Denmark; 2 Section of Metabolic Genetics, Novo Nordisk Foundation Center for Basic Metabolic Research, Faculty of Health and Medical Sciences, University of Copenhagen, Blegdamsvej 3B, DK-2200 Copenhagen N, Denmark; 3 The Children’s Obesity Clinic, Department of Pediatrics, Copenhagen University Hospital Holbæk, Holbæk, Denmark; 4 Center for Clinical Research and Disease Prevention, Bispebjerg and Frederiksberg Hospital, The Capital Region, Copenhagen, Denmark; 5 Department of Clinical Experimental Research, Rigshospitalet, Glostrup, Denmark; 6 Department of Clinical Medicine, Faculty of Health and Medical Sciences, University of Copenhagen, Copenhagen, Denmark; 7 Steno Diabetes Center, Gentofte, Denmark; 8 Faculty of Health Sciences, University of Southern Denmark, Odense, Denmark; 9 Section of Systems Genomics, Department of Bio and Health Informatics, Technical University of Denmark, Kongens Lyngby, Denmark

**Keywords:** Epidemiology, Genetic association, Genetic risk score, Genetics, Insulin resistance, Insulin sensitivity, Obesity, Paediatric obesity, Weight regulation

## Abstract

**Aims/hypothesis:**

A genetic risk score (GRS) consisting of 53 insulin resistance variants (GRS_53_) was recently demonstrated to associate with insulin resistance in adults. We speculated that the GRS_53_ might already associate with insulin resistance during childhood, and we therefore aimed to investigate this in populations of Danish children and adolescents. Furthermore, we aimed to address whether the GRS associates with components of the metabolic syndrome and altered body composition in children and adolescents.

**Methods:**

We examined a total of 689 children and adolescents who were overweight or obese and 675 children and adolescents from a population-based study. Anthropometric data, dual-energy x-ray absorptiometry scans, BP, fasting plasma glucose, fasting serum insulin and fasting plasma lipid measurements were obtained, and HOMA-IR was calculated. The GRS_53_ was examined for association with metabolic traits in children by linear regressions using an additive genetic model.

**Results:**

In overweight/obese children and adolescents, the GRS_53_ associated with higher HOMA-IR (β = 0.109 ± 0.050 (SE); *p* = 2.73 × 10^−2^), fasting plasma glucose (β = 0.010 ± 0.005 mmol/l; *p* = 2.51 × 10^−2^) and systolic BP SD score (β = 0.026 ± 0.012; *p* = 3.32 × 10^−2^) as well as lower HDL-cholesterol (β = −0.008 ± 0.003 mmol/l; *p* = 1.23 × 10^−3^), total fat-mass percentage (β = −0.143 ± 0.054%; *p* = 9.15 × 10^−3^) and fat-mass percentage in the legs (β = −0.197 ± 0.055%; *p* = 4.09 × 10^−4^). In the population-based sample of children, the GRS_53_ only associated with lower HDL-cholesterol concentrations (β = −0.007 ± 0.003 mmol/l; *p* = 1.79 × 10^−2^).

**Conclusions/interpretation:**

An adult-based GRS comprising 53 insulin resistance susceptibility SNPs associates with insulin resistance, markers of the metabolic syndrome and altered fat distribution in a sample of Danish children and adolescents who were overweight or obese.

**Electronic supplementary material:**

The online version of this article (10.1007/s00125-018-4640-0) contains peer-reviewed but unedited supplementary material, which is available to authorised users.

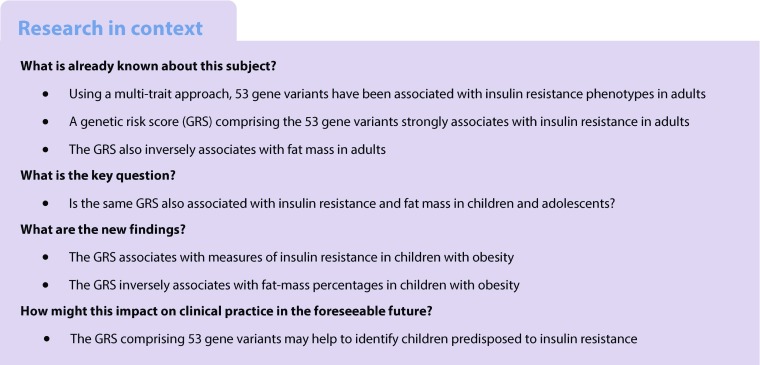



## Introduction

Individuals diagnosed with the metabolic syndrome have an increased risk of developing cardiometabolic diseases such as cardiovascular disease and type 2 diabetes [1]. Insulin resistance (IR), a complex metabolic condition with both genetics and the environment as contributing factors, has been suggested to be the primary mediator of the metabolic syndrome [1]. Obesity, especially visceral obesity [2], is a crucial factor in the development of IR, and the prevalence of obesity is rapidly increasing in both children and adults. In paediatric populations, obesity strongly associates with alterations in glucose metabolism, and impaired glucose metabolism and IR are observed not only in adults, but also in a large fraction of children with obesity [3, 4]. Furthermore, as increased BMI during childhood strongly correlates with increased risk of developing type 2 diabetes in adulthood [5], it is of great importance to identify risk factors potentially influencing or even mediating the link between childhood obesity and adult type 2 diabetes. Examination of factors predisposing to IR in clinical subsets of children who are overweight or obese is thus particularly relevant. If individuals predisposed to IR are identified early in life, targeted measures could potentially delay the development of IR, and thereby potentially the development of cardiovascular disease and type 2 diabetes later in life.

A genetic component of the development of IR is evident from genome-wide association studies in adults [6–8]. Recently, 53 genetic loci associated with IR-related phenotypes, i.e. fasting insulin adjusted for BMI, and circulating concentrations of triacylglycerol and HDL-cholesterol, were identified through an integrative genomic approach [9]. A genetic risk score (GRS) comprising the 53 lead SNPs from the identified loci associated with IR (GRS_53_) [9], as based on measures from a euglycaemic–hyperinsulinaemic clamp, an insulin suppression test and an insulin sensitivity index from a frequently sampled OGTT [10, 11]. Furthermore, the GRS_53_ associated with lower BMI, body fat percentage and leg, arm and gynoid fat mass [9]. GRSs for type 2 diabetes are reported to have a higher predictive value in younger individuals than older ones [12–17], probably due to a higher impact of genetic vs environmental factors in youth. The same concept may hold true for a GRS for IR, which could potentially be used as a clinical tool in the identification of children for whom early intervention might be especially relevant. Previously, a GRS relating to IR based on only ten SNPs was reported to have no association with IR in 1076 children with obesity [18], yet the associations of a GRS comprising the newly identified 53 SNPs in a paediatric population remain unknown.

The aims of this study were therefore to investigate the following in a sample of Danish children and adolescents who were overweight or obese, as well as in a population-based sample: (1) the associations of the GRS_53_ with estimates of IR phenotypes (fasting concentrations of insulin, triacylglycerols and HDL-cholesterol) and HOMA-IR; (2) the SNP-specific effects on these phenotypes; and (3) the associations of the GRS_53_ with other components of the metabolic syndrome and body composition. The GRS_53_ was initially validated in an adult Danish population.

## Methods

### Inter99 study population

Clinical data for adults were obtained from the Inter99 study (ClinicalTrials.gov NCT00289237). The Inter99 study is a population-based, randomised, non-pharmacological intervention study for the prevention of ischaemic heart disease, conducted by the Research Centre for Prevention and Health, Glostrup University Hospital, Glostrup, Denmark. Of 13,016 individuals (aged 30–60 years) randomly selected from the Civil Registration System and invited to participate, 6784 (52%) participated in baseline examinations. Detailed phenotypic characteristics from Inter99 have previously been published [19], and baseline characteristics are presented in Table 1.

**Table 1:** Clinical characteristics of participants

Variable			TDCOB			
	Inter99		TCOCT sample		Population-based control sample	
	*n*	Median (interquartile range)	*n*	Median (interquartile range)	*n*	Median (interquartile range)
Sex (male/female)	2565/2690		306/383	–	266/409	–
Age (years)	5255	45.1 (39.9–50.2)	689	11.65 (9.7–13.9)	675	12.54 (10.1–15.2)
Biochemical measures						
HOMA-IR	5063	1.36 (0.9–2.0)	689	4.34 (2.8–6.4)	675	2.23 (1.7–6.4)
Fasting serum insulin (pmol/l)	5065	34 (23–50)	689	115.00 (77.0–166.5)	675	61.24 (45.5–83.9)
Fasting plasma glucose (mmol/l)	5251	5.4 (5.1–5.8)	689	5.10 (4.9–5.4)	675	4.97 (4.8–5.2)
HbA_1c_ (mmol/mol)	5249	39.9 (36.6–43.2)	686	34.00 (33.0–37.0)	636	35.00 (33.0–36.0)
HbA_1c_ (%)	5249	5.8 (5.5–6.1)	686	5.3 (5.2–5.5)	636	5.4 (5.2–5.4)
Fasting plasma LDL-cholesterol (mmol/l)	5186	3.42 (2.8–4.1)	680	2.50 (2.1–3.0)	635	2.10 (1.8–2.5)
Fasting plasma HDL-cholesterol (mmol/l)	5247	1.40 (1.2–1.7)	680	1.20 (1.0–1.4)	635	1.50 (1.3–1.7)
Fasting plasma total cholesterol (mmol/l)	5246	5.4 (4.8–6.2)	680	4.20 (3.7–4.8)	635	3.90 (3.6–4.5)
Fasting plasma triacylglycerol (mmol/l)	5251	1.0 (0.8–1.5)	680	0.90 (0.7–1.3)	635	0.60 (0.5–0.9)
Anthropometrics						
BMI (kg/m^2^)	5253	25.39 (23.1–28.3)	NA	NA	NA	NA
BMI SDS	NA	NA	689	2.92 (2.5–3.3)	675	0.34 (−0.4–1.0)
WHR	5252	0.85 (0.8–0.9)	664	0.98 (0.9–1.0)	672	0.83 (0.8–0.9)
BP						
Systolic BP (mmHg)	5254	130 (120–140)	NA	NA	NA	NA
Diastolic BP (mmHg)	5253	80 (75–90)	NA	NA	NA	NA
Systolic BP SDS	NA	NA	660	1.55 (0.8–2.6)	642	1.57 (0.8–2.6)
Diastolic BP SDS	NA	NA	660	0.59 (0.1–1.2)	642	0.42 (−0.1–0.9)
DXA						
Fat mass, total (%)	NA	NA	391	43.96 (40.4–47.6)	44	25.26 (22.2–31.5)
Fat mass, torso (%)	NA	NA	391	45.18 (40.5–49.9)	44	20.27 (16.9–28.5)
Fat mass, legs (%)	NA	NA	391	45.63 (42.3–48.8)	44	31.00 (27.5–36.6)
Fat mass, arms (%)	NA	NA	391	46.02 (42.4–50.0)	44	31.76 (24.9–36.4)

#### Anthropometric measurements

While wearing light indoor clothes and no shoes, height (cm) and weight (kg) were measured, and BMI was calculated as weight (kg) divided by height squared (m^2^). Waist and hip circumference were measured in cm, and WHR was calculated as waist measurement (cm) divided by hip measurement (cm).

#### BP

This was measured using a mercury sphygmomanometer.

#### Blood sampling

Blood samples were drawn following a 12 h overnight fast, and measures of insulin, blood glucose, HDL-cholesterol, triacylglycerol and total cholesterol were obtained as previously described [19, 20]. All participants without previously diagnosed diabetes underwent a standardised 75 g glucose OGTT, from which participants were diagnosed with type 2 diabetes according to the WHO 1999 criteria. No individuals with previously diagnosed or screen detected type 2 diabetes were included in the present study.

#### Genotyping

This was performed on 5255 participants from the Inter99 cohort, using the Illumina HumanOmniExpress-24 v1.0_A and HumanOmniExpress-24 v1.1_A (Illumina, San Diego, CA, USA). Genotypes were called using the Genotyping module (version 1.9.4) of GenomeStudio software (version 2011.1; Illumina). Only individuals having a call rate ≥98% were included. Genotypes were phased using Eagle on autosomes and Shapeit on chromosome X and imputed in the Phase 3 1KG and HRC1.1 using the Michigan imputation server (https://imputationserver.sph.umich.edu/index.html) [21]. All variants included in this study were in Hardy–Weinberg equilibrium (*p* > 0.05).

### The Danish Childhood Obesity Biobank study population

Clinical data on Danish children and adolescents was obtained from The Danish Childhood Obesity Biobank (TDCOB; ClinicalTrials.gov NCT00928473). Between March 2007 and March 2013, 1069 children and adolescents (aged 6–18 years) who were overweight or obese were recruited from the Children’s Obesity Clinic, Department of Pediatrics, Copenhagen University Hospital Holbæk as part of the Children’s Obesity Clinic’s Treatment Protocol (TCOCT) [22] (see Table 1 for clinical characteristics). In the following sections, this study sample will be referred to as the TCOCT sample. Overweight was defined as a BMI above the 90th percentile for sex and age according to Danish BMI charts [23] (corresponding to a BMI SD score (SDS) >1.28). All measures included in this study were obtained at the first visit to the clinic, i.e. before treatment initiation. Between September 2010 and March 2013, a population-based sample of 719 children and adolescents (6–18 years) were recruited from local schools and high schools (see Table 1 for clinical characteristics). In the following sections, this study sample will be referred to as the population-based control sample.

#### Anthropometric measurements

With participants wearing light indoor clothes and no shoes, height was measured by a stadiometer (to the nearest 1 mm), and weight was measured on a digital scale (to the nearest 0.1 kg). BMI was calculated as the weight (kg) divided by the height squared (m^2^), and BMI SDS was calculated using the least mean squares method [24] based on a Danish reference [23]. Waist circumference was measured at umbilical level in the upright position after exhalation using a stretch-resistant tape (to the nearest 5 mm). The WHR was calculated as the waist measurement (cm) divided by the hip measurement (cm).

#### BP

Systolic and diastolic BP were measured with an oscillometric device (Omron 705IT; Omron Healthcare, Kyoto, Japan) with the appropriate cuff size, as validated in children [25]. BP was measured three times on the right upper arm after 5 min of rest. An average of the last two measurements was used to calculate systolic and diastolic BP SDS based on sex-, age- and height-specific American references [25].

#### Blood sampling

Blood samples were drawn from an antecubital vein after an overnight fast. Whole-blood HbA_1c_ was analysed on a Tosoh HPLC G8 analyser (Tosoh Corporation, Tokyo, Japan). Plasma glucose was measured on a Dimension Vista 1500 Analyser (Siemens Healthcare, Erlangen, Germany), and serum insulin, plasma cholesterol, plasma HDL-cholesterol and plasma triacylglycerol on a Cobas 6000 Analyser (Roche Diagnostics, Mannheim, Germany).

#### Dual-energy x-ray absorptiometry

Measurements taken using dual-energy x-ray absorptiometry (DXA) included fat mass in the arms, legs, torso and whole body. Measures were performed using a GE Lunar Prodigy (DF+10031 GE Healthcare, Little Chalfont, UK) until 14 October 2009 and thereafter using a GE Lunar iDXA (ME+200,179; GE Healthcare).

#### Genotyping

DNA was extracted at LGC Genomics (Teddington, UK), and samples from all participants (*n* = 1788) were genotyped using the Illumina Infinium HumanCoreExome Beadchip (Illumina, San Diego, CA, USA) using Illumina’s HiScan system at the Novo Nordisk Foundation Center for Basic Metabolic Research’s laboratory, Symbion, Copenhagen, Denmark. Genotypes were called using the Genotyping module (version 1.9.4) of GenomeStudio software (version 2011.1; Illumina). We excluded individuals who were duplicates or ethnic outliers, or had extreme inbreeding coefficients, mislabelled sex or a call rate of <95%, leaving 1618 individuals. Additional genotypes were imputed using the 1000 genomes phase 1 panel using shapeit/IMPUTE2 pipeline (http://mathgen.stats.ox.ac.uk/impute/impute_v2.html) [26, 27], with only genotyped variants that were not significant (*p* > 0.05) in Hardy–Weinberg equilibrium tests. Only variants with a high imputation quality (IMPUTE2 estimated *R*^2^ > 0.95) were kept.

### GRS construction

Genotypes were coded according to the number of IR-increasing alleles based on 53 independent SNPs shown to associate with IR phenotypes (higher fasting insulin concentrations adjusted for BMI, lower HDL-cholesterol concentrations and higher triacylglycerol concentrations) in adults [9] (electronic supplementary material [ESM] Table 1). All genotypes were retrieved from the imputed dataset, and GRS construction was therefore based on genotype dosage information. We constructed an unweighted GRS by summing the number of IR phenotype-increasing alleles. In addition, we constructed a weighted GRS by summing the number of IR phenotype-increasing alleles weighted by the effect size of the variants on fasting insulin concentrations adjusted for BMI, as reported in the validation study in adults [9], and normalised by dividing by the sum of all effects, to make the two GRSs comparable. Similar results were obtained from the two GRSs, and therefore only results from the unweighted GRS (GRS_53_) are reported.

### Statistical analyses

Only children and adolescents from the TDCOB cohort with available information on HOMA-IR were included in our analyses (*n* = 1364). For clinical characteristics of study participants included in the analyses, see Table 1. All statistical analyses were performed with and without the inclusion of participants who had conditions or were receiving medication potentially influencing IR, such as long-term present or prior systemic use of steroid hormones (*n* = 29). Statistical analyses were performed using R software (version 3.1.3; R Foundation for Statistical Computing, Boston, MA, USA). HOMA-IR was calculated as ([fasting plasma glucose (mmol/l)] × [fasting serum insulin (pmol/l)])/135 [28]. LDL-cholesterol was calculated according to the Friedewald formula: [LDL-cholesterol (mmol/l) = total cholesterol (mmol/l) − HDL-cholesterol (mmol/l) − triacylglycerol (mmol/l)/5] [29]. Associations between the GRS and IR, metabolic traits and body composition estimates were examined by linear regression using additive genetic models. Analyses were adjusted for sex and age where indicated, and all analyses of DXA measures were adjusted for type of DXA scanner. Quantitative traits deviating from normal distribution were log-transformed (log_10_) to ensure a normal distribution as assumed in the model. For log-transformed traits, the corresponding *p* values are reported. Furthermore, clinically interpretable effect sizes and SEs from the analyses of untransformed traits are reported. Binominal tests were performed to assess the directionality of SNP-specific effects. Differences in effect sizes between groups were assessed using a standard two-tailed *t*-test with β values and SEs for each group. Correction for multiple testing was performed using a false discovery rate (FDR) of 10% [30]. Values of *p* < 0.05 were considered statistically significant.

### Ethical aspects

Written informed consent was obtained from all participants. If they were younger than 18 years, informed oral consent was given by the participant while the parents provided informed written consent. The study was approved by the Danish Data Protection Agency (REG-06-2014), the Ethics Committee of Region Zealand, Denmark (SJ-104) and the Scientific Ethics Committee of the Capital Region of Denmark (KA98155). The study was performed in accordance with the Declaration of Helsinki 2013 and is registered at ClinicalTrials.gov (NCT00928473 and NCT00289237).

## Results

### Validation of the association between the GRS_53_ and IR phenotypes in Danish adults

As a means of validating the association of the GRS_53_ with IR phenotypes in an adult Danish population, we used data from the Inter99 study population comprising 5255 non-diabetic Danish individuals (aged 30–60 years). The Inter99 population was not part of the study by Lotta et al [9], and Inter99 therefore constitutes an adult study sample suitable for validating the association between the GRS_53_ and IR phenotypes. In the Danish adults, the unweighted GRS_53_ associated with fasting concentrations of insulin adjusted for BMI (β = 0.014 ± 0.003; *p* = 2.25 × 10^−7^), triacylglycerol (β = 0.022 ± 0.003; *p* = 1.14 × 10^−12^) and HDL-cholesterol (β = −0.018 ± 0.003; *p* = 4.86 × 10^−10^) (ESM Table 2) with effect sizes similar to those previously reported [9]*.* Furthermore, the unweighted GRS_53_ associated with HOMA-IR in the Danish adults (β = 0.014 ± 0.003; *p* = 1.45 × 10^−7^) (ESM Table 2). Similar results were obtained for the weighted GRS_53_.

### Association of the GRS_53_ with IR phenotypes in children and adolescents

All statistical tests were performed with and without the inclusion of individuals (*n* = 29) who had conditions or were receiving medication potentially influencing IR, but the results obtained did not differ (data not shown). Only results based on the inclusion of these individuals are thus provided. In the TCOCT sample, the GRS_53_ associated with HOMA-IR (β = 0.109 ± 0.050; *p* = 2.73 × 10^−2^); however, no association was observed in the population-based control sample (*p* = 0.10) (Table 2). The GRS_53_ was inversely associated with HDL-cholesterol in both the TCOCT sample (β = −0.008 ± 0.003 mmol/l; *p* = 1.23 × 10^−3^) and the population-based control sample (β = −0.007 ± 0.003 mmol/l; *p* = 1.79 × 10^−2^) (Table 2). The GRS_53_ did not associate with concentrations of fasting insulin or triacylglycerol in either population (*p* > 0.05) (Table 2). Despite the fact that the observed associations of the GRS_53_ were greater in the TCOCT sample, no statistically significant difference in the effect size of the GRS_53_ in the two groups was identified, as determined by a two-tailed *t* test (Table 2).

**Table 2:** Association between GRS_53_ and IR and related metabolic traits

Variable	TCOCT sample				Population-based control sample				Difference in GRS effect size between groups *p*_*t* test_
	*n*	β ± SE	*p* value	*p* value (FDR 10%)	*n*	β ± SE	*p* value	*p* value (FDR 10%)	
Biochemical measures									
HOMA-IR	689	0.109 ± 0.050	2.73 × 10^−2^*^a,b^	0.09	675	0.021 ± 0.012	0.10^a,b^	0.59	0.22
Fasting serum insulin (pmol/l)	689	2.250 ± 1.114	0.05^a,b^	0.12	675	0.495 ± 0.307	0.11^a,b^	0.59	0.27
Fasting plasma glucose (mmol/l)	689	0.010 ± 0.005	2.51 × 10^−2^*^a,b^	0.09	675	0.003 ± 0.003	0.40^a,b^	0.70	0.35
HbA_1c_ (mmol/mol)	686	0.016 ± 0.028	0.58^b^	0.66	636	0.005 ± 0.025	0.86^b^	0.97	0.67
Fasting plasma LDL-cholesterol (mmol/l)	680	0.007 ± 0.007	0.37^a,b^	0.46	635	0.004 ± 0.005	0.49^a,b^	0.70	0.61
Fasting plasma HDL-cholesterol (mmol/l)	680	−0.008 ± 0.003	1.23 × 10^−3^**^a,b^	8.00 × 10^−3^**	635	−0.007 ± 0.003	1.79 × 10^−2^*^a,b^	0.29	0.68
Fasting plasma total cholesterol (mmol/l)	680	0.003 ± 0.007	0.73^a,b^	0.78	635	−0.002 ± 0.006	0.55^a,b^	0.70	0.54
Fasting plasma triacylglycerol (mmol/l)	680	0.007 ± 0.005	0.23^a,b^	0.34	635	0.001 ± 0.003	0.94^a,b^	0.97	0.46
Anthropometrics									
BM SDS	689	−0.007 ± 0.006	0.26	0.35	675	0.0003 ± 0.009	0.97	0.97	0.54
WHR	664	−6.267 × 10^−5^ ± 6.655 × 10^−4^	0.93^b^	0.93	672	0.0004 ± 0.001	0.48^b^	0.70	0.60
BP									
Systolic BP SDS	660	0.026 ± 0.012	3.32 × 10^−2^*	0.09	642	−0.011 ± 0.012	0.35	0.70	0.17
Diastolic BP SDS	660	0.013 ± 0.008	0.09	0.14	642	−0.007 ± 0.006	0.23	0.70	0.32
DXA									
Fat mass, total (%)	391	−0.143 ± 0.054	9.15 × 10^−3^**^b^	4.80 × 10^−2^*	44	−0.140 ± 0.203	0.49^b^	0.70	1.00
Fat mass, torso (%)	391	−0.116 ± 0.066	0.08^b^	0.14	44	−0.156 ± 0.213	0.47^b^	0.70	0.71
Fat mass, legs (%)	391	−0.197 ± 0.055	4.09 × 10^−4^***^b^	6.40 × 10^−3^**	44	−0.133 ± 0.231	0.57^b^	0.70	0.62
Fat mass, arms (%)	391	−0.116 ± 0.062	0.06^b^	0.12	44	−0.149 ± 0.258	0.57^b^	0.70	0.76

Results are shown for the unweighted GRS. Effect sizes and SEs were calculated using untransformed variables. Values for *p* were calculated using untransformed variables unless otherwise indicated. All DXA scan analyses were adjusted for type of scanner

^a^*p* value calculated using log-transformed (base 10) variables

^b^Analyses adjusted for age and sex

*p < 0.05, **p < 0.01 and ***p < 0.001

### SNP-specific associations with IR phenotypes

**Fig. 1:**
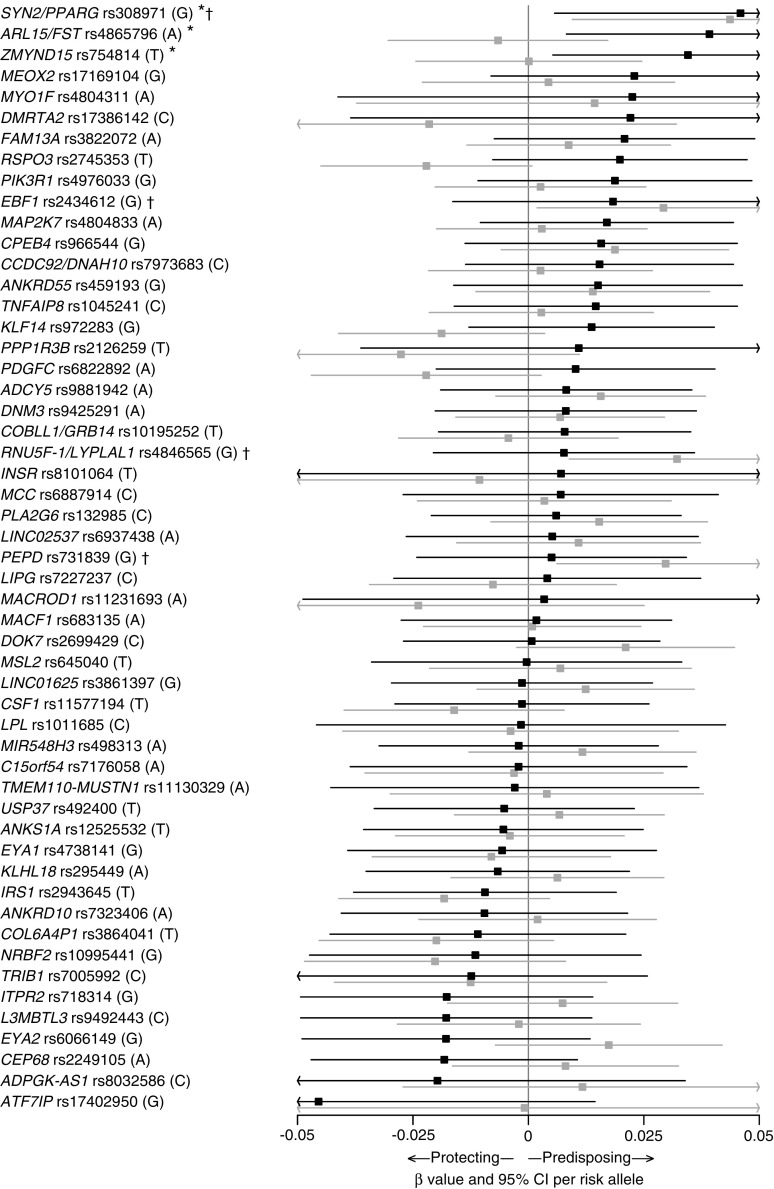
SNP-specific associations with HOMA-IR in children from the TCOCT sample (black lines) and population-based control sample (grey lines). For each SNP, the name of the nearest gene, rs number and risk allele as reported [9] are provided. *p < 0.05 for the association between the given SNP and HOMA-IR in the TCOCT sample. ^†^*p* < 0.05 for the association between the given SNP and HOMA-IR in the population-based sample

When calculating the individual association of each SNP with HOMA-IR, fasting insulin, HDL-cholesterol and triacylglycerol, associations were identified for three, three, five and four SNPs, respectively, in the children with obesity, whereas associations were identified for four, four, four and six SNPs, respectively, in the control population (Fig. 1, ESM Figs 1–3). Consistency of the SNP-specific directionally effect sizes was only observed for HDL-cholesterol in the children with obesity, with 38 out of 53 SNPs showing negative directional effects (*p* = 2.19 × 10^−3^; ESM Table 3). In contrast, consistent directional effects of the included SNPs were observed for all four traits examined in the adult Inter99 population (ESM Table 3).

### Association between the GRS_53_ and metabolic traits

When investigating whether the GRS_53_ associated with additional traits related to the metabolic syndrome, the GRS_53_ associated with higher fasting plasma glucose concentrations (β = 0.010 ± 0.005 mmol/l; *p* = 2.51 × 10^−2^) in the TCOCT sample (Table 2), whereas no association was observed in the population-based control sample (*p* = 0.40) (Table 2). Although no associations between the GRS_53_ and BMI SDS, WHR, HbA_1c_ or diastolic BP SDS were found in either of the populations, an association between the GRS_53_ and the systolic BP SDS was identified in the TCOCT sample (β = 0.026 ± 0.012; *p* = 3.32 × 10^−2^) (Table 2). No difference in the effect size of the GRS_53_ between the two study populations was identified (Table 2).

### Association between GRS_53_ and measures of fat deposition

In the TCOCT sample, we observed an association between the GRS_53_ and lower total fat-mass percentage (β = −0.143 ± 0.054%; *p* = 9.15 × 10^−3^) (Table 2). We also investigated fat-mass percentages for specific parts of the body, such as the arms, legs and torso. In the TCOCT sample, the GRS_53_ associated with reduced leg fat-mass percentage (β = −0.197 ± 0.055%; *p* = 4.09 × 10^−4^) (Table 2). In the population-based control sample, no associations between GRS_53_ and measures of fat deposition were observed, yet the effect size of the GRS_53_ did not differ between the examined study populations, as determined by a two-tailed *t* test (Table 2).

## Discussion

In this study, we sought to investigate whether the associations of a GRS previously associated with IR phenotypes (fasting insulin adjusted for BMI, HDL-cholesterol and triacylglycerol) in adults [9] are already evident during childhood and adolescence. We initially demonstrated that the GRS_53_ also associated with these phenotypes and the HOMA-IR in Danish adults. We then proceeded to examine whether these associations between the GRS_53_ and IR phenotypes could be identified in two samples of Danish children and adolescents: one sample that was population-based, and the other comprising children and adolescents who were overweight/obese, thereby representing a clinical subset with elevated risk of obesity-induced IR. In the TCOCT sample comprising overweight/obese children and adolescents, the GRS_53_ associated with higher HOMA-IR, but no association was identified in the population-based control sample. Previously, the 53 SNPs included in the GRS have all been independently associated with IR in adults [9]. In the current study, however, only a few of the 53 SNPs displayed associations with either HOMA-IR, fasting insulin, HDL-cholesterol or triacylglycerol in both Danish children and adults, a discrepancy which may be due to the larger statistical power of the original study [9]. Although the majority of the included loci exhibited same-directional effect sizes, compared with the reports made in the discovery study [9], formal tests of directional effects only reached statistical significance for HDL-cholesterol in the children with overweight/obesity, and for all the examined traits in the Danish adults. This same-directional effect was more evident in the Danish adult population than the child populations, suggesting that the association of the SNPs may be stronger in adulthood than childhood and may thus be age-dependent. Nevertheless, as the current study has only limited power to detect SNP-specific associations, larger study populations of both children and adults seem necessary to elucidate whether the included SNPs do in fact display age-dependent effects.

In a recent study by Morandi et al comprising 1076 children with obesity (mean age 11.4 years) and 1265 young adults with normal weight (mean age 21.1 years), no associations between a GRS comprising ten IR-associated SNPs and OGTT-derived traits (fasting insulin, fasting glucose and HOMA-IR) were identified [18]. This discrepancy in relation to our results may be explained by the difference in the SNPs included in the GRS. Morandi et al used in their GRS ten SNPs originally chosen by Vassy et al [17] because of their association with HOMA-IR, higher fasting insulin or IR-related traits such as lower HDL-cholesterol or higher triacylglycerol, BMI or WHR in earlier publications. The GRS_53_ used in the current study consists of 53 SNPs used by Lotta et al to create their GRS [9]. By employing a meta-analysis, Lotta et al included up to 188,577 individuals and identified 630 SNPs within 53 loci associating with higher fasting insulin, lower HDL-cholesterol and higher triacylglycerol. The 53 lead SNPs from these loci were then compiled into the GRS. In the same study, the association between the GRS_53_ and IR in adults was validated based on previously obtained measures from a euglycemic–hyperinsulinemic clamp or insulin suppression test in 2764 adults, or by insulin sensitivity index in 4769 individuals [10, 11] . The better validated and higher number of SNPs in the GRS_53_ used in our study may explain why the GRS_53_, in contrast to the earlier publication [18], associates with IR despite our smaller study population.

We found that the GRS_53_ associated with other traits of the metabolic syndrome, namely lower HDL-cholesterol concentrations and higher systolic BP in the TCOCT sample. Interestingly, the GRS_53_ was inversely associated with HDL-cholesterol level in the population-based control children as well. In contrast, there were no associations between the GRS_53_ and higher fasting insulin and triacylglycerol concentrations in either population, even though associations with these traits, together with HDL-cholesterol, were originally used to identify the susceptibility SNPs in adults. Unfortunately, concentrations of fasting plasma lipids and BP were not investigated by Lotta et al [9]. Furthermore, in the TCOCT sample, the GRS_53_ associated with higher fasting plasma glucose, in consistency with the association identified in adults [9]. The GRS_53_ has previously been associated with lower BMI and higher WHR/hip circumference in adults [9]; however, no associations between the GRS_53_ and these traits were identified in either of our child populations.

When examining the DXA-derived measures of fat deposition, the GRS_53_ showed an association with lower total fat percentage and leg fat-mass percentage, corresponding to the findings in adults [9]. IR, lower fat mass and ectopic fat deposition associate with the GRS_53_ in adults [9]. As the GRS_53_ in our study associates with both HOMA-IR and lower fat-mass percentage, it is likely that the GRS_53_ may associate with ectopic lipid deposition, a potential cause of IR, in children as well. A more detailed analysis of lipid accumulation in liver and skeletal muscles is needed to validate this hypothesis.

Although we observed more associations between the GRS_53_ and IR-related phenotypes in the sample of children with obesity, the identified effect estimates of the GRS_53_ in the two groups of children did not differ. With our statistical power, we can therefore not claim that the associations of the GRS_53_ differ between our two populations. Nevertheless, our results still indicate that the GRS_53_ has a greater association in children with obesity, compared with a population-based sample of children. Collectively, our results obtained in Danish children, adolescents and adults suggest that the association of the GRS comprising the 53 SNPs are mediated via metabolic stresses such as obesity and ageing. Our findings indicate a different effect size of the individual SNPs in the GRS_53_ in populations comprising children and adolescents compared with adults. Potentially, the SNP-specific associations are modified by obesity, ageing and other exposures. Our findings correspond well with previous studies reporting age-dependent effects of loci associating with metabolic traits such as BMI and obesity [31–33]. An age-dependent variation of the effect of the individual SNPs in the GRS_53_ complicates the interpretation of data and calls for the identification of age-specific trait loci and subsequent construction of age- and trait-specific GRSs.

Although our results must be validated in larger paediatric study populations, our findings could have clinical implications, as they suggest that children with an increased risk of IR may benefit from preventive and therapeutic lifestyle and/or pharmacological treatment approaches aiming to reduce obesity to prevent the development of IR-associated cardiometabolic risks. Furthermore, our findings indicate that the GRS_53_ has potential as a clinical marker to aid the identification of children with a higher risk of IR than that mediated via obesity alone. In adults, the GRS_53_ strongly associated with increased risk of developing type 2 diabetes [9], yet it remains unknown whether children with a high genetic burden, as assessed by the GRS_53_, also have an increased risk of developing diabetes and/or diabetes-related comorbidities later in life. Future studies examining the associations of the GRS_53_ in larger populations and across a lifespan could potentially help to elucidate whether the GRS_53_ could be used as a clinical tool that would, during childhood and adolescence, already enable the identification of individuals with an increased risk of IR and ultimately type 2 diabetes. As such, the GRS_53_, or even an improved GRS comprising other or additional SNPs with validated strong associations with IR phenotypes in childhood, could potentially be one of the first steps towards personalised intervention programmes aiming to minimise the occurrence of cardiovascular events and preterm death associated with early-onset type 2 diabetes [34].

Our study is limited by the relatively small study sample, which reduces the statistical power. Furthermore, analyses were not adjusted for stage of puberty but only for age. Nevertheless, a very detailed phenotypic characterisation was available for the included study population, enabling the detailed analysis of several traits related to IR and fat deposition. Furthermore, data for the examined traits were available for two populations of children with similar age spans yet different ranges of BMI SDS, enabling an evaluation of the effect of obesity on the effect of the GRS. It should be noted that the two populations of children were selected in different ways: the children with obesity were highly selected according to their BMI and age, whereas the children from the population-based group were selected only according to age. This discrepancy in the selection of study participants may potentially affect our findings.

In conclusion, we investigated whether the GRS_53_ associates with IR phenotypes and HOMA-IR in both children and adolescents who are overweight or obese, and in a population-based control sample. A GRS associating with IR in children could help to identify children predisposed to IR. In overweight or obese children and adolescents, the 53 SNPs cumulatively associate with IR. The results indicate that children who have a genetic predisposition to IR, as assessed by the 53 SNPs, will have a higher risk of developing IR if they become overweight or obese. However, as no difference between the effects size of the GRS_53_ in the two groups of children could be identified, we cannot with certainty conclude that obesity is essential for the association between HOMA-IR and the GRS_53_. This hypothesis needs to be verified in a larger population. The identification of additional SNPs displaying strong associations with IR-related phenotypes during childhood would increase the clinical impact of the GRS_53_ and allow the identification of children predisposed to IR. Treatment strategies targeted against factors important for the development of IR, such as obesity, could be developed specifically for predisposed children. Furthermore, our study showed that fat percentage in the body extremities was inversely associated with GRS_53_ in the children and adolescents who are overweight or obese, suggesting that impaired capacity to store fat in peripheral compartments increases the risk of IR.

## Electronic supplementary material

ESM (PDF 408 kb)

## Data Availability

The datasets generated during and/or analysed during the current study are available from the corresponding author on reasonable request.

## Abbreviations

DXA: Dual-energy x-ray absorptiometry
FDR: False discovery rate
GRS: Genetic risk score
GRS_53_: Genetic risk score comprising 53 SNPs known to associate with insulin resistance-related phenotypes in adults
IR: Insulin resistance
SDS: SD score
TCOCT: The Children’s Obesity Clinic’s Treatment Protocol
TDCOB: The Danish Childhood Obesity Biobank
