# Supplementary material for: An adult-based insulin resistance genetic risk score associates with insulin resistance, metabolic traits and altered fat distribution in Danish children and adolescents who are overweight or obese
Source: Diabetologia. 2018 May 31;61(8):1769–79. doi: 10.1007/s00125-018-4640-0 (PMC6061152; doi:10.1007/s00125-018-4640-0)
Supplement: Supplementary file 1 — (PDF 408 kb) [file 125_2018_4640_MOESM1_ESM.pdf]

Electronic supplementary material

Tables

ESM Table 1. Information on the 53 SNPs included in the GRS_{53}

Chromosome	Position	SNP	Locus name	Risk/Other allele	Risk allele frequency
Chr 8	19830769	rs1011685	*LPL*	C/T	0.90
Chr 2	165513091	rs10195252	*COBLL1 / GRB14*	T/C	0.57
Chr 5	118729286	rs1045241	*TNFAIP8*	C/T	0.71
Chr 10	64868989	rs10995441	*NRBF2*	G/T	0.21
Chr 3	52896605	rs11130329	*TMEM110-MUSTN1*	A/C	0.86
Chr 11	63862362	rs11231693	*MACROD1*	A/G	0.07
Chr 1	110499925	rs11577194	*CSF1*	T/C	0.47
Chr 6	35004569	rs12525532	*ANKS1A*	T/C	0.40
Chr 22	38563471	rs132985	*PLA2G6*	C/T	0.54
Chr 7	15883477	rs17169104	*MEOX2*	G/C	0.30
Chr 1	50815533	rs17386142	*DMRTA2*	C/T	0.92
Chr 12	14571421	rs17402950	*ATF7IP*	G/A	0.06
Chr 8	9185146	rs2126259	*PPP1R3B*	T/C	0.10
Chr 2	65287646	rs2249105	*CEP68*	A/G	0.66
Chr 5	158021791	rs2434612	*EBF1*	G/A	0.21
Chr 4	3479886	rs2699429	*DOK7*	C/T	0.41
Chr 6	127452685	rs2745353	*RSPO3*	T/C	0.54
Chr 2	227098930	rs2943645	*IRS1*	T/C	0.63
Chr 3	47375705	rs295449	*KLHL18*	A/G	0.60
Chr 3	12116370	rs308971	*SYN2 / PPARG*	G/A	0.13
Chr 4	89741019	rs3822072	*FAM13A*	A/G	0.43
Chr 6	139828666	rs3861397	*LINC01625*	G/A	0.37
Chr 3	15185384	rs3864041	*COL6A4P1*	T/C	0.59
Chr 5	55806501	rs459193	*ANKRD55*	G/A	0.75
Chr 8	72469492	rs4738141	*EYA1*	G/A	0.24
Chr 19	8615339	rs4804311	*MYO1F*	A/G	0.94

Chr 19	7970385	rs4804833	*MAP2K7*	A/G	0.40
Chr 1	219721854	rs4846565	*RNU5F-1 / LYPLAL1*	G/A	0.66
Chr 5	53272414	rs4865796	*ARL15 / FST*	A/G	0.70
Chr 2	219349502	rs492400	*USP37*	T/C	0.57
Chr 5	67713996	rs4976033	*PIK3R1*	G/A	0.42
Chr 9	78033919	rs498313	*MIR548H3*	A/G	0.69
Chr 20	45602388	rs6066149	*EYA2*	G/A	0.74
Chr 3	135926372	rs645040	*MSL2*	T/G	0.79
Chr 4	157734425	rs6822892	*PDGFC*	A/G	0.67
Chr 1	39895210	rs683135	*MACF1*	A/G	0.31
Chr 5	112711236	rs6887914	*MCC*	C/T	0.79
Chr 6	43815114	rs6937438	*LINC02537*	A/G	0.74
Chr 8	126528705	rs7005992	*TRIB1*	C/G	0.16
Chr 15	39463917	rs7176058	*C15orf54*	A/G	0.84
Chr 12	26453033	rs718314	*ITPR2*	G/A	0.26
Chr 18	47174429	rs7227237	*LIPG*	C/T	0.78
Chr 19	33898815	rs731839	*PEPD*	G/A	0.34
Chr 13	111627945	rs7323406	*ANKRD10*	A/G	0.30
Chr 17	4656784	rs754814	*ZMYND15*	T/C	0.69
Chr 12	124448973	rs7973683	*CCDC92 / DNAH10*	C/A	0.67
Chr 15	73080817	rs8032586	*ADPGK-AS1*	C/T	0.92
Chr 19	7292869	rs8101064	*INSR*	T/C	0.03
Chr 1	172312519	rs9425291	*DNM3*	A/G	0.44
Chr 6	130398481	rs9492443	*L3MBTL3*	C/T	0.75
Chr 5	173350155	rs966544	*CPEB4*	G/A	0.32
Chr 7	130466604	rs972283	*KLF14*	G/A	0.51
Chr 3	123082166	rs9881942	*ADCY5*	A/G	0.46

SNP rs numbers are provided as reported by Lotta et al [1] along with locus name. SNP positions are based on build GRCh37/hg19. I addition, the risk allele frequencies, as calculated from the TCOCT and control population of children used in the present study, are provided (n = 1618).

ESM Table 2. Association between GRS_{53} and insulin resistance and other metabolic traits in Inter99 (non-diabetic individuals).

	$uGRS_{53}$			$wGRS_{53}$		
	n	β ± SE	*p* value	*n*	β ± SE	*p* value
Biochemical measures						
HOMA-IR	5061	0.014 ± 0.003	1.45×10^{-7}[a]***	5061	0.011 ± 0.003	2.65×10^{-6}[a]***
Fasting serum insulin	5063	0.014 ± 0.003	2.25×10^{-7}[a]***	5063	0.012 ± 0.003	2.62×10^{-6}[a]***
Fasting plasma glucose	5249	0.006 ± 0.003	0.04[a]*	5249	0.004 ± 0.003	0.08[a]
HbA_{1c}	5247	0.006 ± 0.003	0.06[a]	5247	0.003 ± 0.003	0.32[a]
Fasting plasma HDL-cholesterol	5247	-0.018 ± 0.003	4.86×10^{-10}[b]***	5247	-0.014 ± 0.003	3.81×10^{-7}[b]***
Fasting plasma total cholesterol	5244	0.007 ± 0.003	1.60×10^{-2}[a]*	5244	0.008 ± 0.003	5.88×10^{-3}[a]**
Fasting plasma triacylglycerol	5251	0.022 ± 0.003	1.14×10^{-12}[b]***	5251	0.018 ± 0.003	6.04×10^{-7}[b]***
Anthropometrics						
BMI	5253	-0.002 ± 0.003	0.54[b]	5253	-0.002 ± 0.003	0.48[b]
Waist-hip ratio	5250	0.010 ± 0.002	8.25×10^{-7}[a]***	5250	0.009 ± 0.002	1.20×10^{-6}[a]***
Blood pressure						
Systolic BP	5252	0.004 ± 0.003	0.19[a]	5252	0.003 ± 0.003	0.32[a]
Diastolic BP	5251	0.006 ± 0.003	2.50×10^{-2}[a]*	5251	0.007 ± 0.003	7.80×10^{-3}[a]**

Results are shown for the unweighted (uGRS) and weighted GRS (wGRS). Effect sizes, standard errors and *p* values were calculated using inverse normal transformation (SD/per allele).

[a]Analyses were adjusted for age, sex and BMI.

[b]Analyses were adjusted for age and sex.

*$p < 0.05$, **$p < 0.01$ and ***$p < 0.001$.

ESM Table 3. Directionality of SNP-specific effects on selected traits.

	Inter99		TDCOB			
			TCOCT sample		Population-based control sample	
Trait	Ratio of same-directional effects	*p* value	Ratio of same-directional effects	*p* value	Ratio of same-directional effects	*p* value
HOMA-IR	42/53	2.25×10^{-5}***	31/53	0.27	31/53	0.27
Fasting serum insulin	41/53	8.17×10^{-5}***	31/53	0.27	31/53	0.27
Fasting plasma HDL-cholesterol	42/53	2.25×10^{-5}***	38/53	2.19×10^{-3}**	33/53	0.10
Fasting plasma triacylglycerol	45/53	2.37×10^{-7}***	26/53	1.00	25/53	0.78

Direction of effects was classified based on the direction of effect reported in Lotta et al [1].

*$p < 0.05$, **$p < 0.01$ and ***$p < 0.001$.

Figures

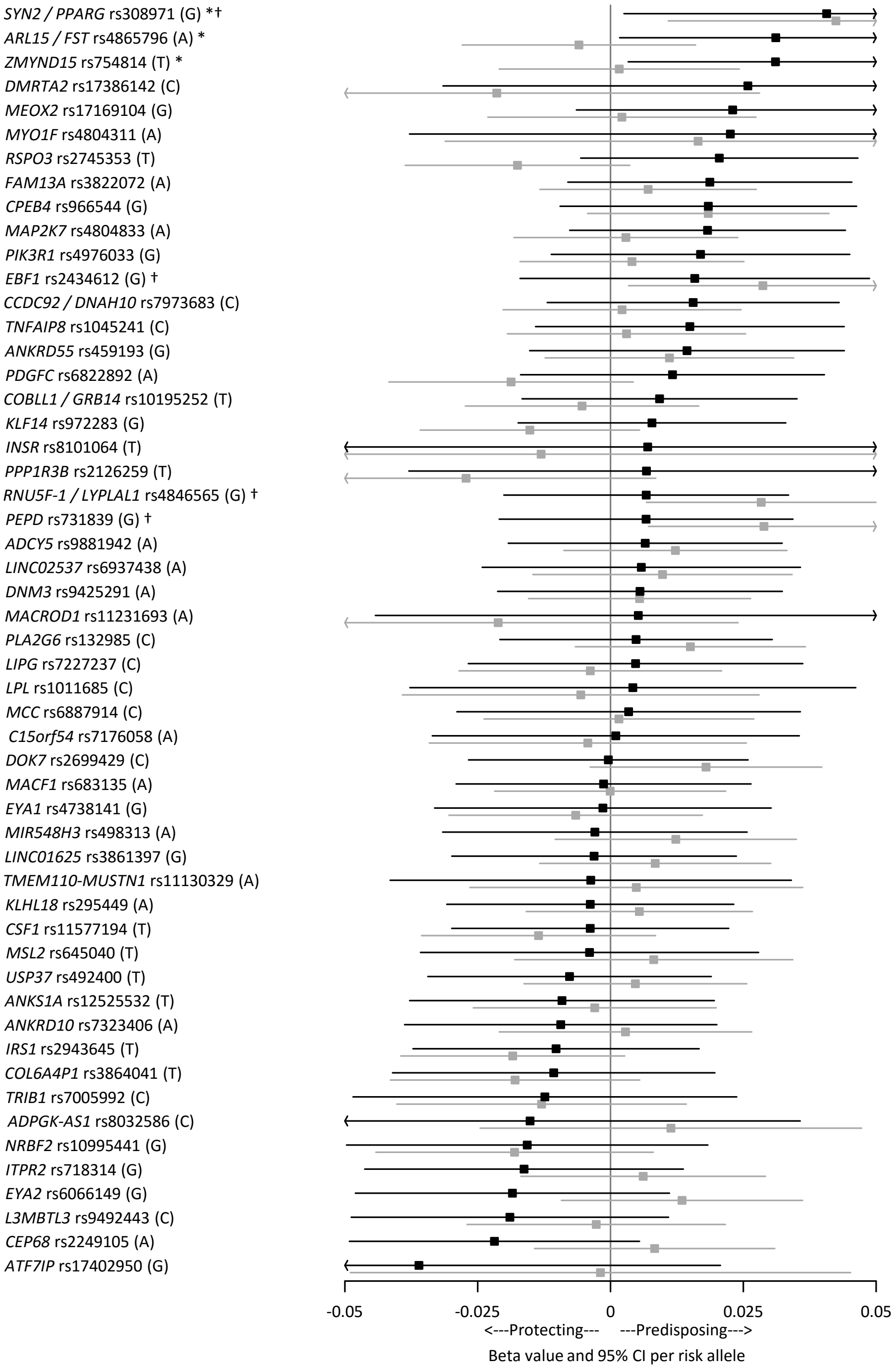

ESM Fig. 1. SNP-specific associations with fasting serum insulin concentrations in children from the TCOCT sample (black lines) and population-based control sample (grey lines). For each SNP, name of nearest gene, rs number, and risk allele as reported [1] are provided. *$p < 0.05$ for the association between the given SNP and fasting serum insulin in the TCOCT sample. †$p < 0.05$ for the association between the given SNP and fasting serum insulin in the population-based sample.

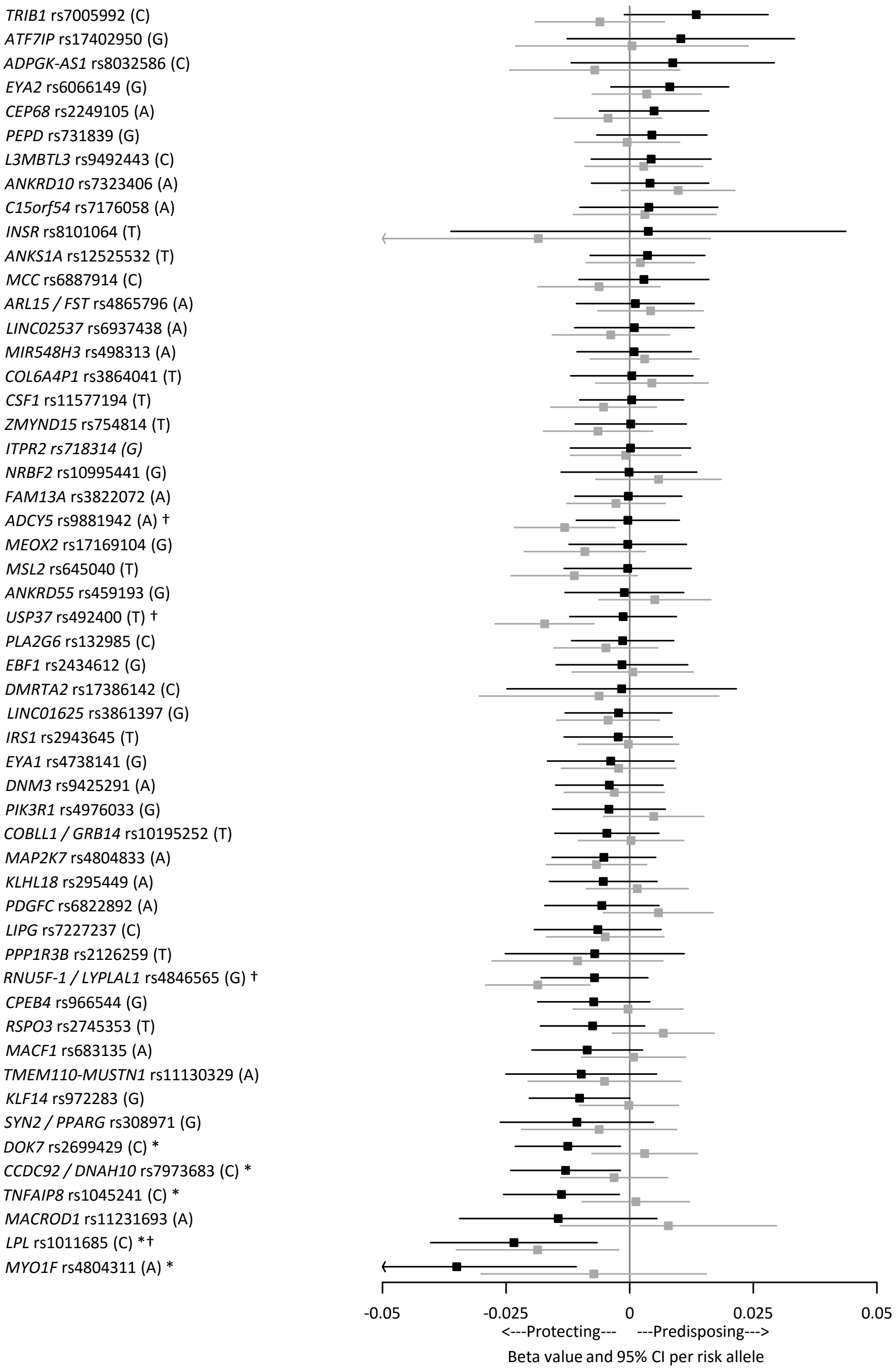

ESM Fig. 2. SNP-specific associations with plasma HDL-cholesterol concentrations in children from the TCOCT sample (black lines) and population-based control sample (grey lines). For each SNP, name of nearest gene, rs number, and risk allele as reported [1] are provided. *$p < 0.05$ for the association between the given SNP and plasma HDL-cholesterol in the TCOCT sample. †$p < 0.05$ for the association between the given SNP and plasma HDL-cholesterol in the population-based sample.

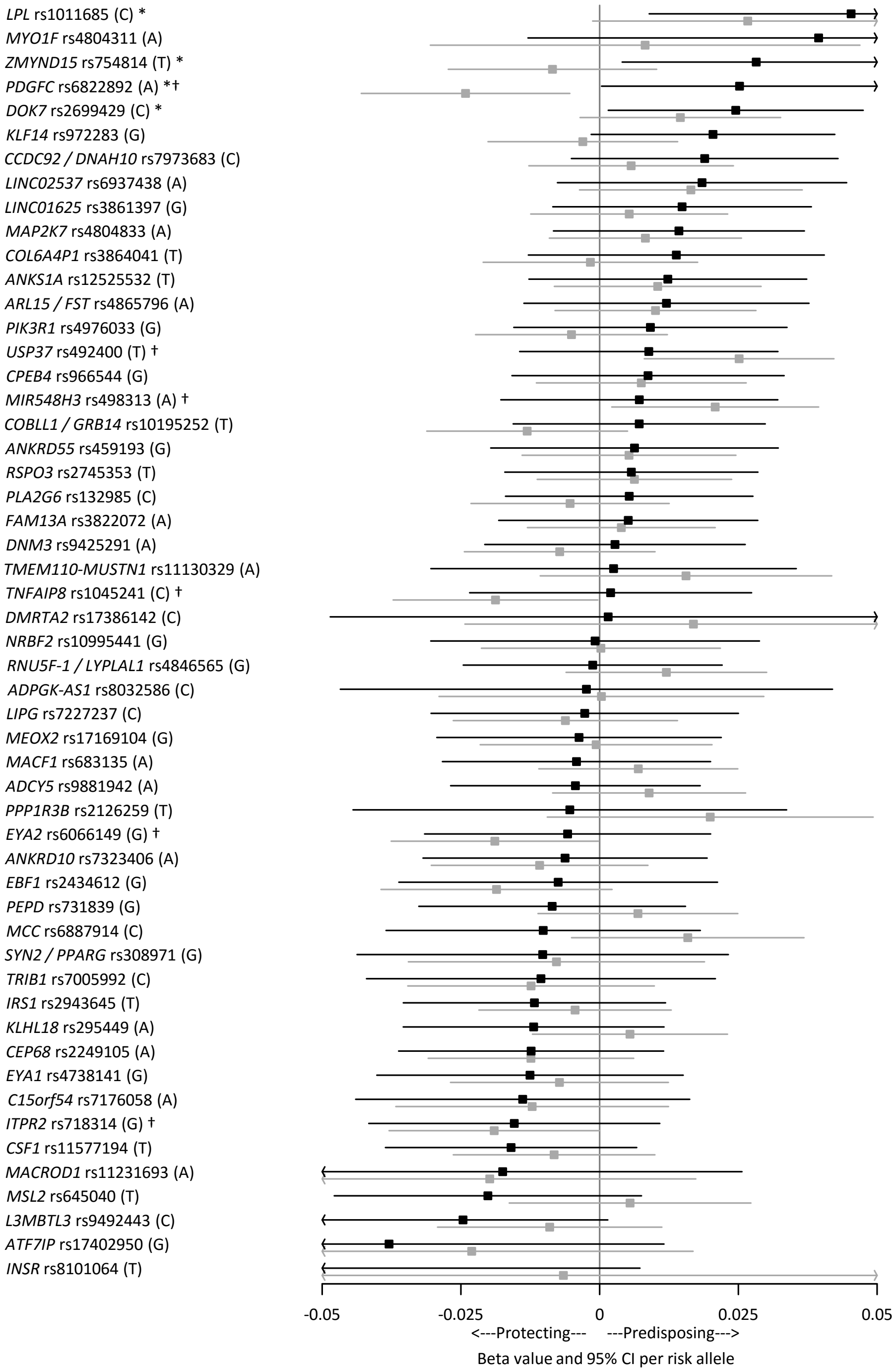

ESM Fig. 3. SNP-specific associations with plasma triacylglycerol concentrations in children from the TCOCT sample (black lines) and population-based control sample (grey lines). For each SNP, name of nearest gene, rs number, and risk allele as reported [1] are provided. *$p < 0.05$ for the association between the given SNP and plasma triacylglycerol in the TCOCT sample. †$p < 0.05$ for the association between the given SNP and plasma triacylglycerol in the population-based sample.

References

1. Lotta LA, Gulati P, Day FR, et al (2017) Integrative genomic analysis implicates limited peripheral adipose storage capacity in the pathogenesis of human insulin resistance. Nat Genet 49:17–26.
